# Immunomodulatory Effects of Probiotics on Cytokine Profiles

**DOI:** 10.1155/2018/8063647

**Published:** 2018-10-23

**Authors:** Md. Abul Kalam Azad, Manobendro Sarker, Dan Wan

**Affiliations:** ^1^Hunan Province Key Laboratory of Animal Nutritional Physiology and Metabolic Process, Key Laboratory of Agroecological Processes in Subtropical Region, Institute of Subtropical Agriculture, Chinese Academy of Sciences, National Engineering Laboratory for Pollution Control and Waste Utilization in Livestock and Poultry Production, Changsha, Hunan 410125, China; ^2^University of Chinese Academy of Sciences, Beijing 100049, China; ^3^Biomass Energy Engineering Research Centre, School of Agriculture and Biology, Shanghai Jiao Tong University, 800 Dongchuan Road, Shanghai 200240, China; ^4^Key Laboratory of Urban Agriculture (South), Ministry of Agriculture, 800 Dongchuan Road, Shanghai 200240, China; ^5^Department of Food Engineering and Technology, State University of Bangladesh, Dhaka 1205, Bangladesh; ^6^Academician Workstation of Hunan Baodong Farming Co., Ltd., Hunan 422001, China

## Abstract

Probiotics confer immunological protection to the host through the regulation, stimulation, and modulation of immune responses. Researchers have shifted their attention to better understand the immunomodulatory effects of probiotics, which have the potential to prevent or alleviate certain pathologies for which proper medical treatment is as yet unavailable. It has been scientifically established that immune cells (T- and B-cells) mediate adaptive immunity and confer immunological protection by developing pathogen-specific memory. However, this review is intended to present the recent studies on immunomodulatory effects of probiotics. In the early section of this review, concepts of probiotics and common probiotic strains are focused on. On a priority basis, the immune system, along with mucosal immunity in the human body, is discussed in this study. It has been summarized that a number of species of* Lactobacillus* and* Bifidobacterium *exert vital roles in innate immunity by increasing the cytotoxicity of natural killer cells and phagocytosis of macrophages and mediate adaptive immunity by interacting with enterocytes and dendritic, Th1, Th2, and Treg cells. Finally, immunomodulatory effects of probiotics on proinflammatory and anti-inflammatory cytokine production in different animal models have been extensively reviewed in this paper. Therefore, isolating new probiotic strains and investigating their immunomodulatory effects on cytokine profiles in humans remain a topical issue.

## 1. Introduction

Probiotics are living microorganisms that confer several health benefits when administrated in adequate amounts to the host [1, 2]. Adhering to human intestines, probiotics stimulate, regulate, and modulate various different functions, including digestion, metabolism, epithelial innate immunity, competitive exclusion of pathogens, and brain-gut communication [3, 4]. Gut microorganisms produce several nontoxic metabolites and play important roles in nutritional and clinical applications [5–7]. Therefore, the microecology of the gastrointestinal tract, consisting of intestine, microbiota, and nondigestible food within the tract, is crucial for probiotic action in the host.

Probiotics act symbiotically by fermenting nondigestible food, known as prebiotics, for their energy and exert elite properties including antipathogenicity, antiobesity, and diabetic, antidiabetic, anti-inflammatory, anticancer, and angiogenic activities and efficacy on the brain and central nervous system [8]. However, the functions of probiotics can be classified as metabolic, protective, and trophic [9], since the trophic role has garnered attention in studies of immunomodulation. Typically, the immune system in vertebrates can be divided into innate and adaptive immunity. Innate immunity is a nonspecific defense mechanism exerting immediate or near-immediate responses to the presence of pathogens in body. On the other hand, adaptive immunity is highly specific and is able to destroy individual invading pathogens in vertebrates. Besides, a pathogen-specific long-lasting protective memory enables the adaptive immune system to attack and destroy pathogens when reencountered [10]. Lymphocytes, especially B cells and T cells, exert adaptive immune responses by recognizing antigens with their specific receptors.

In the last few years, probiotics have been extensively studied and reported, with humoral, cellular, and nonspecific immunity modulation, as well as promoting the immunological barrier [11, 12]. Probiotics have been evaluated for* in vivo* effects, such as increased peripheral immunoglobin production, stimulation of IgA secretion, and decreased proinflammatory cytokine production [13]. It has been reported that homogenates prepared from several probiotics, including* Lactobacillus rhamnosus GG*,* Lactobacillus acidophilus*,* Lactobacillus delbrueckii* sub sp.* bulgaricus*,* Bifidobacterium lactis*, and* Streptococcus thermophiles,* have the ability to suppress the proliferation of mononuclear cells [14]. It has also been reviewed that* Bifidobacterium bifidum* has a significant effect in enhancing antibody responses to ovalbumin, whereas* B. breve* has an increased humoral immune response after stimulation with IgA [15].

However, published articles regarding the immune responses of probiotics are few in number, while a number of research articles have focused on the metabolic actions of probiotics. In the treatment of various diseases, including inflammation, intestinal bowel diseases, and colon cancer, there is an urgent need to study probiotic strains and their effects on immune modulation. In this review article, particular attention has been paid to probiotics and their immunomodulatory effects on cytokine profiles in terms of pro- and anti-inflammatory cytokines in the host.

## 2. Background and Concept of Probiotics

In scientific and clinical research,* Lactobacillus* (*L. acidophilus*,* L. casei*,* L. salivarius*, and* L. lactis*) and* Bifidobacterium* (*B. bifidum* and* B. lactis*) are commonly found in healthy intestines and are noted as being prominent probiotics [2, 15] having different culture conditions, variability, and diverse characteristics, including sensitivity to low pH, gastric juice, pancreatic and intestinal fluids, bile acid, intestinal or respiratory mucus, adherence to intestinal cells, and interactions with other pathogenic microorganisms in the intestine [7, 8, 16]. Probiotics have been reported to produce a number of nonviable metabolic byproducts, such as bacteriocins, organic acids, acetaldehydes, diacetyl, ethanol, and hydrogen peroxide, which are nontoxic, nonpathogenic, and resistant to enzyme systems in mammals, and have been remarked on as an alternative to antibiotics due to their biological activity and inhibitory properties toward pathogenic microbes in the host [17, 18].

In the gut, probiotic microorganisms act symbiotically and modulate immunity [5, 6, 19]. Several studies have reported that probiotics also produce antioxidants (glutathione) and stimulate activity in reducing oxidative stress. Probiotic microorganisms, including* L. acidophilus* NCDC14 and* L. casei *NCDC19, inhibit lipid peroxidation and decrease streptozotocin- (STZ-) induced oxidative damage in pancreatic tissues of rats [20]. According to Sengul et al. [21], probiotic strains* L. delbrueckii *subsp.* bulgaricus* A13 and* L. delbrueckii* subsp.* Bulgaricus *B3 have a significant effect in reducing oxidative stress in colitis. A probiotic supplement containing freeze-dried beneficial strains, namely,* L. acidophilus*,* L. casei*,* L. rhamnosus*,* L. bulgaricus*,* B. breve*,* B. longum*, and* S. thermophiles*, has been found to increase the plasma total glutathione (GSHt) significantly [22]. However, the health benefits of probiotic microorganisms include prevention of infectious diseases of the intestinal and urinary tracts, prevention of diarrhea, reductions in allergy symptoms, serum cholesterol concentration, blood pressure, stimulation and modulation of the immune system, modulation of gene expression (cytokines, in particular), regression of tumors, and reduction in cocarcinogen production [23]. Researchers are now shifted to discerning deeper understanding of whether probiotics exert health benefits; proposed reaction mechanisms include inhibitory effects of lactate on pathogens, production of short chain fatty acids, lowering the production of toxic substances contributing to pathologies such as inflammatory bowel disease (IBD), and adherence of microbes to the gut through controlling water flow from the blood serum to the intestinal lumen [24]. Most common probiotic microorganisms are presented in Table 1.

In the small intestine, probiotic microorganisms ferment nondigestible carbohydrates, such as fructooligosaccharide, oligofructose, inulin, galactose, and xylose-containing oligosaccharides from various natural sources, including vegetables, fruits, and grains, and readily fulfill their energy requirements [25]. Common prebiotics used in the preparation of synbiotics and their natural sources are summarized in Table 2.* In vivo*,* Lactobacillus casei* has been found to produce significant amounts of GSHt, GSH, and -SH free groups by fermenting inulin, whereas physiological stress results in lower GSH concentrations with large amounts of oxidative stress markers [26]. Verma and Shukla [27] revealed that* L. rhamnosus* and* L. acidophilus *lead to the formation of large quantities of antioxidants, mainly GSH, with inulin being a nondigestible carbohydrate in the small intestine. In another study by Kavitha et al. [28], increased GSH concentrations have been reported due to the combined effects of insulin, pioglitazone, and probiotics in STZ-induced diabetic rats. In a very recent study, the effects of* Lactobacillus plantarum* HII11 are remarkable in preventing adrenomedullin- (ADM-) mediated colon cancer in the rat [29].

## 3. Human Immune System

In the human gut, M cells present in Peyer's patches are crucial due to their capacity to transport macromolecules, antigens, and microorganisms and inert particles from the lumen into the lymphoid tissue by adsorptive endocytosis. In addition, enterocytes and M cells may uptake such as macromolecules, antigens, and microorganisms through a transepithelial vesicular transport mechanism. When antigenic molecules cross the intestinal barrier, they stimulate the innate and adaptive immune systems in the body [56].

### 3.1. Innate and Adaptive Immunity

Humans may come into contact with millions of pathogenic organisms through ingestion, inhalation, and many other ways, while the innate immune system plays a vital role in preventing infection by specific pathogen. Innate immunity is recognized as a first-line defense system against pathogens and can remember previous encounters while attacking again. Phagocytic cells, including neutrophils, monocytes, macrophages, and NK cells, enable this first-line defense system against pathogenic microorganisms in the human body, which destroys pathogens and protects from the corresponding infection. These key players are not specific in recognizing their targets, unlike adaptive immune responses. However, this first-line defense system largely depends on the number of phagocytic cells and proteins, which then activate the adaptive immune response in vertebrates through the activation of antigen-presenting cells (APCs) [57, 58].

On the other hand, development of adaptive immune responses to a new pathogen in the body is comparatively slower than innate immune responses. Usually, lymphocytes (B and T) are key players in the adaptive immune response, which exerts more effective immune responses, having specific antigen receptors, namely, the B cell receptor (BCR) for B cells and T cell receptor (TCR) for T cells [59]. Furthermore, antigen receptors of each naive lymphocyte possess unique specificity. Interestingly, B cells contribute to adaptive immunity, through the secretion of antibodies, known as humoral immunity, while T cells contribute cell-mediated immunity through subdivision into T helper cells (CD4+, called Th) and cytotoxic T cells (CD8+) [60]. It has been well known that B cells recognize specific antigens via BCRs, whereas CD8+ cells recognize antigens as peptide/MHC class I complexes, and CD4+ cells recognize antigens as peptide/MHC class II complex [61]. Once ACPs are activated, T cells proliferate and differentiate into CD8+ T cells and CD4+ T helper cells. Furthermore, CD8+ T cells convert into cytotoxic T lymphocytes (CTLs), whereas CD4+ T helper cells activate and regulate macrophages and B cells to respond to the adaptive immune system.

### 3.2. Mucosal Immunity

**Figure 1:**
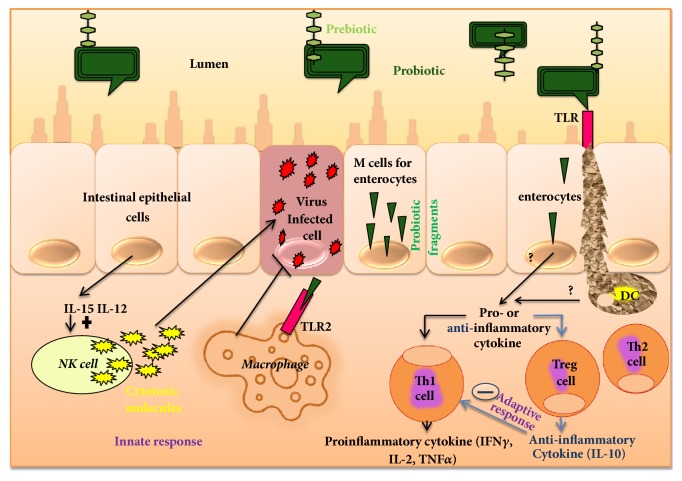
Immunomodulation of probiotics.

The mucosal immune system is very specific in protecting the whole inner surface, involving the oral-pharyngeal cavity, respiratory tract, gastrointestinal (GI) tract, and urogenital tract, as well as the exocrine glands in the human body. The mucosal immune system exerts similar features and anatomical organization for whole inner surface, although organs have different locations in the human body. The GI immune system can represent the mucosal immune system, as shown in Figure 1, while three major compartments, namely, the epithelial layer, lamina propria (LP), and mucosal-associated lymphoid tissue (MALT), are reported to be involved in the GI tract [62, 63]. Lymphoid tissue in the GI tract consists of Peyer's patches, which are characterized by follicle-associated epithelium (FAE) and are distributed in the intestinal epithelium and in secretory sites within the mucosa. However, the epithelial layer and lamina propria are battlefronts, while MALTs act as headquarters and initiate adaptive immune responses. APCs in Peyer's patches capture immunoglobulin A (IgA) antigen from epithelium and microfold cells. Usually, T cells become activated after antigen recognition, and, finally, APCs migrate antigen to lymphoid follicles, lymphoid tissue (LP), and mesenteric lymph nodes. IgA exerts first-line immune defense in the mucosal immune defense with two isotypes of IgA, one being IgA1 in the small intestine and the other being IgA2 in the colonic mucosa produced by B cells [64]. Nevertheless, activated T cells differentiate into effector cells and ensure the integrity of the mucosal barrier and GI environment. However, an application of* Bifidobacterium bifidum *R0071,* Bifidobacterium infantis* R0033, and* Lactobacillus helveticus* R0052 was reported to boost the immunity of infants with changes in salivary immunoglobulin A (SIgA) and the digestive system [65]. Probiotic fermented milk containing* L. casei* DN 114001was found to be effective for gut mucosal immunity in a BALC/c mouse model, with an increased number of T and IgA+B lymphocytes, macrophages, and cells from the nonspecific barrier (goblet cells), while IgA+B was also reported to activate the transcriptional factor NFAT, which is also a nuclear factor of activated T cells [66]. Dogi et al. [67] introduced nonpathogenic Gram-positive and Gram-negative bacteria in some animal models, while only Gram-positive strains, including* L. acidophilus* (strains CRL 1462 and A9) and* L. casei* (CRL 431), have been reported to increase TLR-9 expression.

## 4. Immunomodulation of Cytokine Profiles

Immunomodulatory effects and clinical health benefits of probiotics have been attractive in the treatment of various degenerative diseases. Researchers are now concentrating on identifying the elite properties of probiotics, and some of these include effects on immunity, such as antipathogenicity, antiobesity and diabetic, anti-inflammatory, anticancer, antiallergic, and angiogenic activities and result in effects on the central nervous system (CNS), while efficacy largely depends on the mechanism of action. A number of studies have reported basic molecular mechanisms, such as enhanced IgA secretion, production of cytokines, production of antibacterial substances, enhanced tight junctions of the intestinal barrier against intercellular bacterial invasion, and competition with new pathogenic microorganisms for enterocyte adherence, by which probiotics regulate intestinal epithelial health, although the immunomodulatory effects of probiotics are not the same in every individual and largely depend on environment and epigenetic interactions with the host. In the immunomodulation, probiotic antigenic fragments, such as cell wall compounds, have the ability to cross the intestinal epithelial cells and M cells in Peyer's patches and to then modulate the innate and adaptive immune responses in the body, as illustrated in Figure 1 [68].

The immunomodulatory effect of probiotics is attributed to the release of cytokines, including interleukins (ILs), tumor necrosis factors (TNFs), interferons (IFNs), transforming growth factor (TGF), and chemokines from immune cells (lymphocytes, granulocytes, macrophages, mast cells, epithelial cells, and dendritic cells (DCs)) [69] which further regulate the innate and adaptive immune system [70]. It has been reported that cell wall components of* Bifidobacteria* and* Lactobacilli*, such as lipoteichoic acid, stimulate NO synthase, which is potential in pathogen-infected cell death mechanism (NO) presented by macrophages through TNF-*α* secretion. In addition, two surface phagocytosis receptors (Fc*γ*RIII and tool-like receptor (TLR)) are also upregulated by NO [61, 71]. Probiotics have been reported to interact with enterocytes and dendritic, Th1, Th2, and Treg cells in the intestine and to modulate the adaptive immunity into pro- and/or anti-inflammatory action. Studies with BALB/c (20–30 g) inbred mice and Fisher-344 inbred rats demonstrated that* Lactobacillus paracasei* subsp.* Paracasei* DC412 strain and* L. acidophilus* NCFB 1748 induced early innate immune responses and specific immune markers through phagocytosis, polymorphonuclear (PMN) cell recruitment, and TNF-alpha (TNF-*α*) production [72]. In another experimental animal model involving BALB/c mice, oral administration of* L. casei* favored rapid activation of immune cells and produced a higher number of specific markers such as CD-206 and TLR-2 cells [73], while TLRs improve the immunological defense mechanism in terms of pro- and anti-inflammatory cytokine production upon the detection of foreign objects [74].

### 4.1. Pro- and Anti-Inflammatory Cytokines

**Figure 2:**
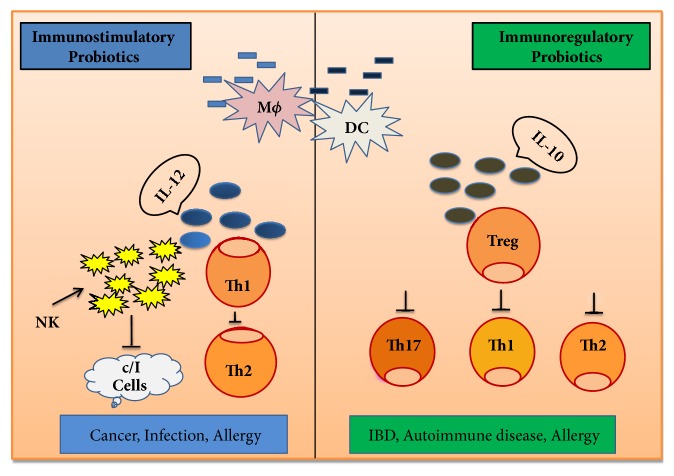
Mechanism of immune regulation by probiotics.

Probiotic strains have a significant influence on the gut barrier by stimulating B cells for the production of IgA. In* in vitro* studies with enterocyte cells (HT-29, caco-2, and dendritic cells derived from PBMC), probiotics have been reported to influence cytokine production by APCs, which initiates adaptive responses. Cytokines also enhance the defense system against invasion by bacterial, fungal, viral, and any pathogenic components. A number of researchers studying the immune system in animal models found that the importance of cytokines lies in binding to specific receptors on cell membrane and in triggering cellular cascades for the induction and improvement, as well as inhibition of several cytokine-regulated genes in the nucleus [75, 76]. The inflammatory process depends on proinflammatory and anti-inflammatory cytokines, where the anti-inflammatory cytokine, interleukin-10 (IL-10), is produced by monocytes, T cells, B cells, macrophages, natural killer cells, and dendritic cells, which inhibit proinflammatory cytokines, chemokines, and chemokine receptors, responsible for intestinal inflammation. A mechanism of immune regulation, involving two distinct categories probiotics (immunostimulatory and immunoregulatory), is shown in Figure 2. Immunostimulatory probiotics have the ability to act against infection and cancer cells, inducing IL-12 production, which activates NK cells and develops Th1 cells. These probiotics also act against allergy through a balance between Th1 and Th2. On the other hand, immunoregulatory probiotics have been characterized with IL-10 and Treg cell production, which results in decreases in allergy, IBD, autoimmune diseases, and inflammatory responses [77].

In an* in vitro* study with Caco-2 cells [78], proinflammatory cytokines (IL-1*β*, IL-8, and TNF-*α*) were induced by* Lactobacillus sakei*, whereas* Lactobacillus johnsonii* influenced the production of TGF-*β* (anti-inflammatory). It has been revealed that IL-6 favors the clonal expansion of IgA B lymphocytes and stimulates the production of antibodies such as IgM, IgG, and reduced secretion of IgE [79]. In addition, anti-inflammatory cytokines, such as IL-4, IL-5, IL-6, IL-10, and IL-13, are produced by Th2 cells, DCs, monocytes, B cells, and Tregs and induce adaptive immune response in the body [80]. In an earlier study, Borruel et al. [81] cultured intestinal mucosa of Crohn's disease patient with nonpathogenic bacteria including* Lactobacillus casei, Lactobacillus bulgaricus, Lactobacillus crispatus*, and* Escherichia coli *to investigate bacterial modulating effects on cytokine responses. Considering a significant reduction in the proinflammatory cytokine TNF-*α* in inflamed mucosa cultured with* L. casei *and* L. bulgaricus*, the authors noted that probiotics interact with immunocompetent cells and modulate the production of proinflammatory cytokines. In an interleukin-10-deficient mouse model,* Lactobacillus salivarius* and* Bifidobacterium infantis* have been used to evaluate their impact on the immune system of hosts in terms of mucosal and systemic cytokine profiles [82]. Significant reductions in interferon-*γ* (INF-*γ*) and TNF-*α* by Peyer's patch lymphocytes and proinflammatory cytokine production by spleen cells were found in probiotic-treated mice. The administration of a mixture of* L. paracasei* and* L. reuteri* to IL-10-deficient mice infected with* Helicobacter hepaticus *resulted in reductions in mucosal proinflammatory cytokines and, consequently, reduced the development of colitis [83]. Yan and Polk [84] found that* Lactobacillus rhamnosus* GG plays a vital role in activating antiapoptotic Akt/protein kinase B and in the inhibition of proapoptotic factors through the p38 MAPK pathway. Recently, Karamese et al. [85] administrated a mixture of* Lactobacillus* and* Bifidobacterium* species to rats for evaluation of the immunomodulatory effects of probiotics, where modulation or regulation of immune responses is evident through the upregulation of IL-10 (an anti-inflammatory cytokine) and the downregulation of TNF-*α* and IL-6 (proinflammatory cytokines). It has also been reported that application of probiotics leads to significant increases in IgA and IgG concentrations in rats, although this is dose-dependent.

Probiotics also have potential as immunomodulators with the ability to interact with epithelium and DCs, monocytes/macrophages, and lymphocytes. Borruel et al. [81] cultured mucosal samples from Crohn's disease patients with* Escherichia coli, Lactobacillus casei, Lactobacillus bulgaricus, *and* Lactobacillus crispatus* and reported that* Lactobacillus casei* and* Lactobacillus bulgaricus* significantly reduced the production of the proinflammatory cytokine TNF-*α* through interaction with immunocompetent cells. The oral administration of lactic acid bacteria, such as* Lactobacillus casei, L. acidophilus, L. rhamnosus, L. delbrueckii subsp. bulgaricus, L. plantarum, Lactococcus lactis, *and* Streptococcus thermophiles,* increases the number of IgA-producing cells associated with lamina propria in mucosa and this effect is dose-dependent. It has been reported that most of the lactic acid bacteria assayed induced inflammatory immune responses, although none of them are unable to induce cytotoxicity mechanisms [86]. Livingston et al. [87] treated bone-marrow-derived dendritic cells (BMDCs) with* Lactobacillus reuteri* 100-23, which were then incubated with splenic T cells from ovalbumin T cell receptor transgenic mice. Anti-inflammatory cytokine IL-10, induced by BMDCs, resulted in lower IL-2 production and increased TGF-*β* production. Different* Lactobacillu*s and* Bifidobacterium* strains demonstrate the potential to trigger epithelial cell expression of IL-10, TGF-*β*, and IL-6 and further stimulate the immunoglobulin production (IgA). Probiotic strains stimulate the immunoglobulin receptors of intestinal epithelial cells [88]. Various animal models suggest that the effects of probiotics on the immunomodulation of cytokines are strain-specific; therefore, combinations of different probiotic strains are beyond the scope of further study to treat inflammation-associated tissue damage and gastrointestinal inflammation in humans.

## 5. Conclusions

Probiotic treatment is a promising research arena in the medical sciences, since probiotics alone, or together with prebiotics, have potential in the modulation of gut microbiota and immune responses in the host. However, a number of scientific reports are identical in terms of the role of probiotics in preventing obesity, inflammatory diseases, and cancer. The immunomodulatory effects of probiotics have gained much attention for the treatment of degenerative and other diseases caused by pathogenic microorganisms. Probiotics have a positive influence on the innate immunity, exerting several antiviral properties. Furthermore, it has been established that probiotics increase gut barrier functions by stimulating B cells and by influencing cytokine production, which initiates adaptive responses in the host body, although there are insufficient research publications regarding how probiotics induce immunomodulatory effects in the treatment of inflammation. It is also urgent to understand cytokine secretion by Th2 cells, DCs, monocytes, B cells, and Tregs in order to establish new strains of probiotics. Further studies can be suggested to determine the precise action of probiotics on inflammation because these findings will be key routes in the medical sector and for better human health.

## Figures and Tables

**Table 1:** Most used probiotic microorganisms.

Probiotic genera	Probiotic strains	References
Lactobacillus	*L. acidophilus, L. amylovorus, L. bulgaricus, L. crispatus, L. casei, L. gasseri, L. helveticus, L. johnsonii, L. pentosus, L. reuteri, L. paracasei, L. plantarum, L. rhamnosus*	[17, 18, 30]
Bifidobacterium	*B. animalis, B. breve, B. infantis, B. bifidum, B. lactis, B. catenulatum, B. longum, B. adolescentis*	[31–34]
Enterococcus	*Enterococcus faecium*	[35]
Streptococcus	*Streptococcus thermophilu*	[36]
Lactococcus	*Lactococcus lactis, L. lactis, L. reuteri, L. rhamnosus, L. casei, L. acidophilus, L. curvatus, L. plantarum*	[37]
Bacillus	*Bacillus clausii, B. coagulans, B. subtilis, B. laterosporus*	[38, 39]
Pediococcus	*Pediococcus acidilactici, P. pentosaceus*	[40]
Propionibacterium	*P. jensenii, P. freudenreichii*	[30]
Streptococcus	*Streptococcus sanguis, S. oralis, S. mitis, S. thermophiles, S. salivarius*	[36]
Bacteroides	*Bacteroides uniformis*	[41]
Enterococcus	*Enterococcus faecium*	[35]
Peptostreptococcus	*Peptostreptococcus productus*	[39]
Escherichia	*Escherichia coli Nissle 1917*	[38]
Faecalibacterium	*Faecalibacterium prausnitzii*	[42]
Akkermansia	*A. muciniphila*	[41]
Saccharomyces	*Saccharomyces cerevisiae, S. boulardi*	[43]

**Table 2:** A list of common prebiotics for the preparation of synbiotics^*∗*^.

Prebiotics	Sources	References
Fructooligosaccharides	Fructooligosaccharides Onion, Leek, Asparagus, Chicory, Jerusalem artichoke, Garlic, Wheat, Oat	[44]
Inulin	Agave, banana, burdock camas, chicory, coneflower, costus, elecampane, globe artichoke, dandelion, Jerusalem artichoke, jicama, wild yam, mugwort root, yacon, garlic, onion	[45]
Isomaltooligosaccharides	Miso, soy, sauce, sake, honey	[46]
Lactulose	Skim milk	[47]
Lactosucrose	Milk sugar	[48]
Galactooligosaccharides	Lentil, human milk, chickpea/hummus, green pea, lima bean, kidney bean	[49, 50]
Soybean oligosaccharides	Soybean	[51]
Xylooligosaccharides	Bamboo shoot, milk, honey	[34]
Fructooligosaccharides	Onion, chicory, garlic, asparagus, banana, artichoke	[52]
Arabinoxylan	Bran of grasses	[53]
Arabinoxylan oligosaccharides	Cereals	[54]
Resistant starch-1,2,3,4	Beans, legumes, starchy fruits and vegetables, whole grains	[55]

^*∗*^This information was reproduced from Kerry et al. [8].
